# First cytogenetic information on four checkered beetles (Coleoptera, Cleridae)

**DOI:** 10.3897/CompCytogen.v14i4.55358

**Published:** 2020-10-27

**Authors:** Atılay Yağmur Okutaner

**Affiliations:** 1 Kırşehir Ahi Evran University, Department of Anthropology, Kırşehir, Turkey Kırşehir Ahi Evran University Kırşehir Turkey

**Keywords:** Chromosome, Cleridae, Coleoptera, cytogenetic, *
Tilloidea
*, *
Trichodes
*

## Abstract

The karyotypes of four species of Cleridae (Coleoptera): *Trichodes favarius* (Illiger, 1802), *Trichodes quadriguttatus* Adams, 1817, *Trichodes reichei* (Mulsant et Rey, 1863), and *Tilloidea transversalis* (Charpentier, 1825) were reported for the first time with this study. The chromosome numbers of these four species were determined as 2n = 18, sex chromosome system Xy_p_, and all chromosomes were metacentric (the except y chromosome). Together with this study, the chromosome data of only 17 species are available in this family. It is remarkable that all of them display the same chromosome number and similar karyotypes. This may make the effect of karyotypical features important in interpreting the evolutionary process of Cleridae.

## Introduction

The Cleroidea containing 16 families and including approximately 10,000 taxonomically defined species is an important superfamily of Coleoptera (Gimmel et al. 2019). After Melyridae, Cleridae is the second largest Cleroid family with almost 3700 species and 350 genera in 13 subfamilies described so far (Opitz 2010; Bulak et al. 2012; Gunter et al. 2013; Gerstmeier 2018). Cleridae are widespread in all continents (except for the Antarctic) and has the highest diversity in the tropics (Gunter et al. 2013). Former analyses of phylogenetic and taxonomic relationships of Cleridae were especially based on morphology (Gerstmeier and Eberle 2011; Opitz 2012; Gunter et al. 2013). Therefore, these relationships were generally determined according to morphological characters with traditional classification systems. The molecular phylogeny of the family is extensively discussed in Gunter et al. (2013).

The data given by chromosomal characters may help to understand the evolutionary relationships of species or higher taxa. Karyological data from the studies in recent years present important findings of genetic structure, life cycle, ecological characteristics, evolution, taxonomy, and phylogeny of insects (Shaarawi and Angus 1991; Gokhman and Kuznetsova 2006). For those reasons, karyotypic features may be referable as a taxonomic character in solving taxonomic problems, assessing relationships, and phylogenetic classification. (Dobigny et al. 2004; Gokhman and Kuznetsova 2006; Miao and Hua 2017).

Although the Cleroidea have a large representative and wide distribution area, only 18 species (13 Cleridae, 5 Melyridae) of the superfamily have been cytogenetically studied so far. The 13 species of Cleridae in five genera (*Enoclerus* Gahan, 1910, *Priocera* Kirby, 1818, *Thanasimus* Latreille, 1806, *Trichodes* Herbst, 1792, and *Necrobia* Olivier, 1795) display monotypic chromosome number as “2n = 18”, the basic sex chromosome system for Coleoptera as Xy_p_, and metacentric/submetacentric morphology for all chromosomes (Smith and Virkki 1978; Schneider et al. 2007; Mendes-Neto et al. 2010).

This study was carried out to support cytogenetic data of the family Cleridae. The chromosomal first data belonging to four species, *Trichodes favarius* (Illiger, 1802), *Trichodes quadriguttatus* Adams, 1817, *Trichodes reichei* (Mulsant et Rey, 1863), and *Tilloidea transversalis* (Charpentier, 1825) were given in this study.

## Material and methods

The localities of collected adult specimens are as follows: 16 *Trichodes favarius* (Illiger, 1802): Hıdırbey village of Samandağ county in Hatay province, 36°8'19"N, 35°58'49"W; 13 *T. quadriguttatus* Adams, 1817: Göksun county in Kahramanmaraş province 37°59'50"N, 36°31'50"W; 8 *T. reichei*: Sıddıklı town in Kırşehir province 39°7'55"N, 33°54'57"W and 14 *Tilloidea transversalis* (Charpentier, 1825): Kesikköprü town in Kırşehir province 38°57'39"N, 34°11'48"W (Leg: A.Y. Okutaner). The specimens were identified by Hüseyin Ozdikmen and were stored in Zoology Lab of Kırsehir Ahi Evran University.

Living beetles were transferred to the laboratory. The gonads and midguts were dissected and isolated from abdominal contents with the aid of a stereomicroscope microscope. The chromosomal preparation procedure was performed according to the method described by Rozek (1994) with partial modifications. The chromosomal preparation procedure in this study was based on the method described by Rozek (1994) with some modifications. The tissues were treated 15–30 min at room temperature with a hypotonic solution containing 1% sodium citrate and 0.005% w/v colchicine. Tissue samples were transferred to cryotubes including 3:1 ethanol: acetic acid solution and stored in the freezer. Each treated sample was placed on a clean slide and disintegrated lightly. With the subsequent addition of the acetic acid: distilled water (1:1) solution, another slide was firmly covered over this slide. These slides were immediately frozen in liquid nitrogen and uncoupled to be stained in 4% Giemsa solution.

The chromosomes of females were obtained only from *Trichodes favarius*. Meiotic chromosome sets of all species were obtained from testis tissues. The chromosome sets fixed on the slides were photographed at 100X magnification with Olympus BX53F microscope equipped with a camera. Chromosome measurements were calculated in terms of µm using the “ImageJ” program with the “levan” plug-in. The chromosome measurements were made from different meiosis metaphase plates of each species and the ideograms were formed with the average for these measurements.

## Results and discussion

**Figure 1.**
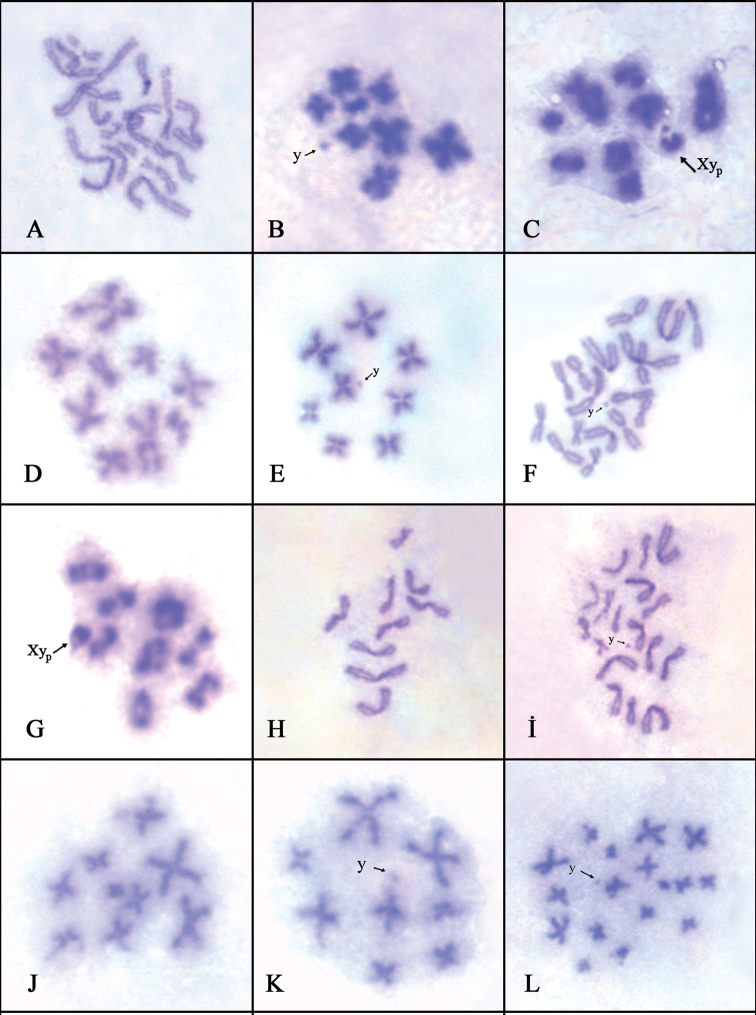
**A** Female Mitotic metaphase of *Trichodes favarius***B, C** male meiotic metaphases of *Trichodes favarius* (**B** meiosis II; **C** meiosis I) **D, E** male meiotic metaphases of *Trichodes quadriguttatus* (**D, E** meiosis II) **F** male mitotic metaphase of *Trichodes quadriguttatus***G, H** male meiotic metaphases of *Trichodes reichei* (**G** meiosis I; **H** meiosis II) **I** male mitotic metaphase of *Trichodes reichei***J, K** male meiotic metaphases of *Tilloidea transversalis* (**J, K** meiosis II) **L** male mitotic metaphase of *Tilloidea transversalis*.

**Figure 2.**
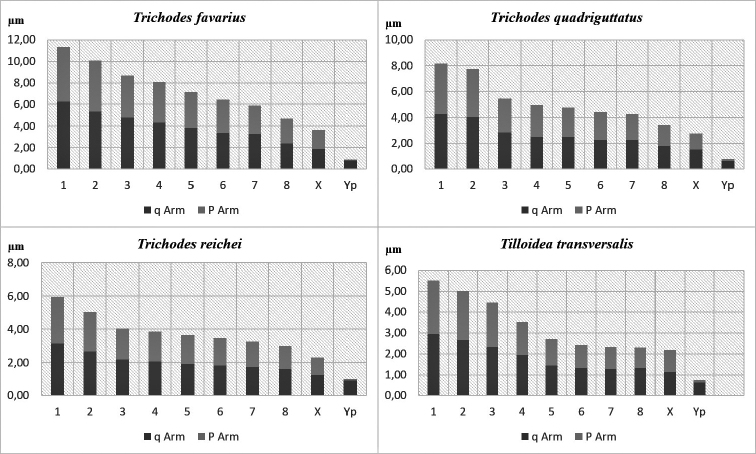
Ideograms of the haploid chromosomes.

The number of the diploid chromosome complement was determined as 2n = 18 and the sex chromosome system as Xy_p_ for each species: *Trichodes favarius*, *Trichodes quadriguttatus*, *Trichodes reichei*, and *Tilloidea transversalis*. The males of these four species display n = 8 + Xy_p_ meioformula. Their chromosome sets (autosomes and X chromosomes) consist of metacentric chromosomes except for subtelocentric y chromosome. Sex chromosome system (association of Xy_p_) in meiosis I, and the presence of y chromosome in meiosis II were clearly demonstrated (Figs 1, 2).

The idiogram shows that the first two chromosome pairs of the species belonging to the genus *Trichodes* are larger than others and a gradual decrease in size in the karyotype of *Tilloidea transversalis* (Fig. 2).

In the previous literature, there is cytogenetic information of only 13 checkered beetles (2 subfamilies, 5 genera). Additionally, cytogenetic data of 4 different species were presented for the first time in this study. After all given data, the diploid chromosome numbers have been presented as 2n = 18 and the sex chromosome system as Xy_p_ of all these 17 Cleridae species. However, four species of Melyridae have observed different chromosome numbers and two different sex chromosome systems XO and Xyp, the chromosome morphologies of these four species are metacentric except for the y chromosome as similar to the Cleridae (Table 1).

**Table 1.** The chromosome data of the Cleridae and Melyridae.

Taxa	Haploid Formula	Diploid Number/Formula	Citations
** Cleridae **			
*Thanasimus dubius* (Fabricius, 1777) (Clerinae)	8+Xy_p_		Smith (1950)
*Trichodes nutalli* (Kirby, 1818) (Clerinae)	8+Xy_p_		Smith (1953)
*Enoclerus nigripes rujiventris* (Spinola, 1844) (Clerinae)	8+Xy_p_	18	
*Enoclerus* sp. (Clerinae)	8+Xy_p_	18	Smith (1960)
*Trichodes ornatus* (Linsley et MacSwain, 1943) (Clerinae)	8+Xy_p_	18	
*Thanasimus formicarius* (Linnaeus, 1758) (Clerinae)	8+Xy_p_	18	Virkki (1960)
*Trichodes apiarius* (Linnaeus, 1758) (Clerinae)	8+Xy_p_	18	
*Enoclerus* sp. (Clerinae)	8+Xy_p_		Virkki (1963)
*Priocera spinosa* (Fabricius, 1801) (Clerinae)	8+Xy_p_		
*Enoclerus moestus* (Klug, 1842) (Clerinae)	8+Xy_p_	18	Smith and Virkki (1978)
*Thanasimus* undatulus (Say, 1835) (Clerinae)	8+Xy_p_		
*Necrobia ruficollis* (Fabricius, 1775) (Corynetinae)	8+Xy_p_	18	Yadav and Dange (1989)
*Necrobia rujipes* (De Geer, 1775) (Corynetinae)	8+Xy_p_	18	
*Trichodes favarius* (Illiger, 1802) (Clerinae)	8+Xy_p_	18	This Study
*Trichodes quadriguttatus* Adams, 1817 (Clerinae)	8+Xy_p_	18	
*Trichodes reichei* (Mulsant et Rey, 1863) (Clerinae)	8+Xy_p_	18	
*Tilloidea transversalis* (Charpentier, 1825) (Tillinae)	8+Xy_p_	18	
** Melyridae **			
*Endeodes collaris* LeConte, 1853 (Malachiinae)		18+X0	Smith and Virkki (1978)
*Collops* sp. (Malachiinae)		16+X0	
*Hoppingiana hudsonica* LeConte 1866 (Dasytinae)		12+Xy_p_	
*Astylus variegatus* (Germar, 1824) (Melyrinae)		16+Xy_p_	Schneider at all (2007)
*Astylus antis* (Perty, 1830) (Melyrinae)	8+X_p or_ y_p_	16+Xy_p_	de Oliveira Mendes-Neto et al. (2010)

Diploid chromosome number 20 and sex chromosome system Xyp are considered ancestral cytogenetic features of Coleoptera, especially the Polyphaga (Smith and Wirkki 1978). According to the limited number of previous studies, it can be said that 2n = 18 chromosome numbers formed by decreasing the ancestral chromosome set (2n = 20) and Xy_p_ sex chromosome system belonging to Cleridae family are quite conservative.

Although it shows variation in the family Melyridae, the numerical changes of chromosomes may not have an important role in the karyotypic evolution of the family Cleridae. Except for the Y chromosome, the metacentric/submetacentric form of all chromosomes may have created a balance for the karyotype of the species. The absence of acrocentric and telocentric chromosomes can reduce the possibility of new centric fusions such as Robertsonian Translocation (Schubert 2007; Chmátal et al. 2014). On the other hand, being resistant to mechanism of chromosome aberration such as chromosome breaks and euploidy may also have created chromosome number stability in the evolutionary process of the family.

In all these respects, the stability of the chromosome set of the family Cleridae is quite remarkable. If these results can be supported by expanding further studies, the cytogenetic features of Cleridae would be very useful taxonomic and evolutionary characters.

## References

[B1] Bulak Y Yıldırım E Gerstmeier R (2012) Contribution to the knowledge of the Cleridae (Coleoptera) fauna of Turkey. Entomofauna 33(23): 325–332.

[B2] Chmátal L Gabriel SI Mitsainas GP Martínez-Vargas J Ventura J Searle JB Schultz RM Lampson MA (2014) Centromere strength provides the cell biological basis for meiotic drive and karyotype evolution in mice. Current biology 24(19): 2295–2300. 10.1016/j.cub.2014.08.017 25242031 PMC4189972

[B3] de Oliveira Mendes-Neto E Vicari MR Campaner C Nogaroto V Artoni RF Almeida MC (2010) Cytogenetic analysis of Astylus antis (Perty, 1830) (Coleoptera, Melyridae): Karyotype, heterochromatin and location of ribosomal genes. Genetics and Molecular Biology 33(2): 237–243. 10.1590/S1415-47572010005000050 21637476 PMC3036849

[B4] Dobigny G Ducroz JF Robinson TJ Volobouev V (2004) Cytogenetics and cladistics. Systematic Biology 53(3): 470–484. 10.1080/10635150490445698 15503674

[B5] Gerstmeier R Eberle J (2011) Definition and Revision of the Orthrius-group of genera (Coleoptera, Cleridae, Clerinae). ZooKeys 92: 35–60. 10.3897/zookeys.92.1157 PMC3084544 21594111

[B6] Gerstmeier R (2018) Checklist of the checkered beetles of Namibia (Coleoptera, Cleridae). Namibian Journal of Environment 2: 7–21.

[B7] Gimmel ML Bocakova M Gunter NL Leschen RAB (2019) Comprehensive phylogeny of the Cleroidea (Coleoptera: Cucujiformia). Systematic Entomology 44: 527–558. 10.1111/syen.12338

[B8] Gokhman VE Kuznetsova VG (2006) Comparative insect karyology: current state and applications. Entomological Review 86(3): 352–368. 10.1134/S0013873806030110

[B9] Gunter NL Leavengood JM Bartlett JS Erıc G Chapman EG Cameron S (2013) A molecular phylogeny of the checkered beetles and a description of Epiclininae a new subfamily (Coleoptera: Cleroidea: Cleridae). Systematic Entomology 38: 626–636. 10.1111/syen.12019

[B10] Miao Y Hua BZ (2017) Cytogenetic comparison between *Terrobittacus implicatus* and *Bittacus planus* (Mecoptera: Bittacidae) with some phylogenetic implications. Arthropod Systematics & Phylogeny 75(2): 175–183. Opitz W (2012) Classification, Natural History, and Evolution of Tarsosteninae (Coleoptera: Cleridae) Part I: Generic Composition of the Subfamily and Key and Phylogeny of Genera. Psyche 2012: 1–35. 10.1155/2012/752402

[B11] Rozek M (1994) A new Chromosome preparation technique for Coleoptera (Insecta). Chromosome Research 2(1): 76–78. 10.1007/BF01539458 8162325

[B12] Schneider MC Carraro BP Cella DM Matiello RR Artoni RF Almeida MC (2007) *Astylus variegatus* (Coleoptera, Melyridae): Cytogenetic study of a Population exposed to agrochemical products. Genetics and Molecular Biology 30(3): 640–645. 10.1590/S1415-47572007000400023

[B13] Schubert I (2007) Chromosome evolution. Current Opinion in Plant Biology 10: 109–115. 10.1016/j.pbi.2007.01.001 17289425

[B14] Shaarawi FA Angus RB (1990) A chromosomal investigation of five European species of Anacaena Thomson (Coleoptera: Hydrophilidae). Insect Systematic and Evolution 21(4): 415–426. 10.1163/187631290X00319

[B15] Smith SG (1950) The cytotaxonomy of Coleoptera. The Canadian Entomologist 82: 58–68. 10.4039/Ent8258-3

[B16] Smith SG (1953) Chromosome numbers of Coleoptera. Heredity 7: 31–48. 10.1038/hdy.1953.3

[B17] Smith SG (1960) Chromosome numbers of Coleoptera. Canadian Journal of Genetics and Cytology 2: 66–88. 10.1139/g60-007

[B18] Smith SG Virkki N (1978) Animal Cytogenetics. V. 3, Insecta 5. Coleoptera. Gebrüder Borntraeger, Berlin, 366 pp.

[B19] Virkki N (1960) Cytology of male meiosis in certain European forest beetles of the families Scolytidae, Cleridae, and Anobiidae. Annales Academiae Scientiarum Fennicae / A / 4B 49: 1–18.

[B20] Virkki N (1963) On the cytology of some Neotropical Cantharoids (Coleoptera). Academiae Scientiarum Fennicae / A / 4B 65: 1–17.

[B21] Yadav JS Dance MP (1989) On the cytology of two species of *Necrobia* (Oliv.) (Coleoptera: Cleridae). Genome 32: 165–167. 10.1139/g89-424

